# Exposure to solar UV radiation of Polish teenagers after the first COVID-19 lockdown in March–April 2020

**DOI:** 10.1007/s00484-022-02337-8

**Published:** 2022-08-01

**Authors:** A. Czerwińska, J. Krzyścin

**Affiliations:** Institute of Geophysics, Polish Academy of Sciences, Warsaw, Poland

**Keywords:** UV radiation, Erythema, Vitamin D, COVID-19

## Abstract

In Poland, schools were closed from March to June 2020 due to the COVID-19 epidemic. During the lockdown (March–April), everyone was advised to stay at home. From May, students were allowed to spend time outdoors. We examine their exposure to solar UV radiation during the period of virtual learning at schools (May–June), vacations (July–August) and the first month of typical learning (September). Primary and high school students aged 12–18 completed a questionnaire on the details of their outdoor activities and the weather at the exposure site. A total of 146 anonymous questionnaires were registered for the study. The survey responses provided input to a radiative transfer model to estimate erythemal and vitamin D doses obtained by teenagers during outdoor activities. The results from 48% of the questionnaires indicated that students’ exposure exceeded 1 minimal erythema dose (MED) during the day. Corresponding doses of sun-synthesized vitamin D, in excess of 1000 international units (IU) and 2000 IU, were found in 77% and 66% of the surveys, respectively. Only 12% of the teenagers declared that they use sunscreen. The overexposure (> 1 MED) increased with age. It was found in 72% and 26% of surveys among the students aged 17–18 and 12–14, respectively. Teenagers seem to have tried to compensate for the lack of sunlight during the lockdown by engaging in outdoor activities permitted since May. While those activities could have improved their vitamin D levels, they also put them at a higher risk of developing erythema.

**Supplementary Information:**

The online version contains supplementary material available at 10.1007/s00484-022-02337-8.

## Introduction

Ultraviolet radiation (UVR), which is only a small part of the solar radiation reaching the ground, is of particular importance to human health. Overexposure to UVR causes a number of diseases such as skin cancer, cataract, oxidative stress and immunosuppression (Modenese et al. 2018; Lucas et al. 2019). Nevertheless, low exposure can lead to vitamin D deficiency (Holick 2017, 2020). Recently, several articles have examined the relationship between vitamin D deficiency and the severity of COVID-19 disease (Weir et al. 2020; Whittemore 2020; DeLuccia et al. 2021; Oristrell et al. 2022; Sidiropoulou et al. 2021; Walrand 2021).

The deficit of vitamin D in adolescents has been a widely discussed issue for the past decade (Gonzalez-Gross 2011Al-Othman et al. 2012; Vierucci et al. 2013; Farrar et al. 2016; Płudowski et al. 2016 Kunz et al. 2018). The serum concentration of 25-hydroxyvitamin D (25(OH)D), a vitamin D status indicator, is often below the optimal or even suboptimal norm. The problem can be addressed not only in the countries located in Northern Europe (Poland, UK, Sweden, Germany), but also in more southern countries, such as Italy, Greece, Spain and Hungary. In Germany, even a change in the recommendation of vitamin D supplementation in 2012 (from 200 to 800 international units (IU)) had no effect on the vitamin D status in adolescents (Kunz et al. 2018). In Poland, the recommended dose for supplementation in adolescents (11 to 18 years of age) ranges from 800 to 2000 IU, when it is not possible to obtain a sufficient dose from sun exposure (Rusińska et al. 2018). In addition, from May to September, healthy people are advised to expose as much of their body as possible for 15 min from 10 a.m. to 3 p.m.

In a typical school year, students do not have time for outdoor activities on weekdays. Classes at school last until 2–3 p.m., and thus, after school, spending time outside may not be sufficient to get the recommended vitamin D intake from skin synthesis. School trips, PE lessons and free time on weekends are most likely the only opportunities that Polish pupils have to obtain the recommended dose of vitamin D from sunlight. Unfortunately, in most Polish schools, children are not allowed to leave the building during school breaks.

While vitamin D deficiency among teenagers is well documented, patterns of behaviour in the sun may pose another problem (Fernández-Morano et al. 2017; Hubbard et al. 2020; Liew and Cust 2021). The occurrence of melanoma is associated not with chronic outdoor exposure, but with episodes of high-intensity UV radiation (Gandini et al. 2005; Dennis et al. 2008). The number of sunburns in childhood and adolescence may determine the risk of melanoma occurrence in adulthood. Therefore, preventing erythema in this age group is of particular importance.

Our project “UV radiation and Vitamin D_3_”, which was one of the BRITEC (Bringing Research Into the Classroom) pilots as a part of the “ERASMUS + project” action, aimed to combine citizen science with school education. The description of this project can be found at the website www.britec.igf.edu.pl. The idea was to involve scientists, teachers and students in jointly solving the important problem of overexposure to UV radiation. Unfortunately, by the time the project was supposed to begin (Spring 2020), the COVID-19 pandemic had started. To avoid the spreading of the disease, a number of restrictions were introduced in Poland. One of them was the “stay at home” policy, i.e. a policy which prohibited students from going outside and forced the students to continue their education virtually. From May, students were allowed to go outside, but schools were closed until the end of June 2020. The project had to be adapted to account for the emerging COVID-19 restrictions. This study aims to model the erythemal and pre-vitamin D doses obtained by adolescents after the COVID-19 lockdown, based on information about their outdoor activities and on-site weather provided by the students involved in the project to assess the risk associated with the overexposure to solar radiation.

## Methodology

### Citizen science: online surveys

From May to September 2020, volunteers among students completed a questionnaire regarding the details of their outdoor activities and the weather at the exposure site. Their observations and personal data (age, weight and height) were placed anonymously in an online survey and sent to the researcher via Google forms. In early April, during online training, scientists gave volunteers instructions on how to fill in the questionnaire. Information provided by the students was converted into inputs to a radiative transfer model to estimate erythemal and vitamin D doses obtained during their outdoor activities. Only total column ozone (TCO_3_) data was collected from external online resources.

Volunteers, aged from 12 to 18 years, sent 173 anonymous surveys, among them 146 passed the quality check, i.e. comprised responses to all questions in a proper format. Surveys from three primaries and four high schools were available for further studies (Table 1). Based on geographic location, age, weight and height, we found that 87 teenagers responded. Each questionnaire was treated independently despite the possibility of sending several questionnaires by the same person.

**Table 1:** List of the schools that participated in the BRITEC project

School	Latitude (° N)	Longitude (° E)	Elevation above the sea level (m)
Primary school in Rząska	50.10	19.84	252.4
Primary school number 62, Poznań	52.45	16.89	90.8
Primary school number 12, Katowice	50.25	19.04	265.8
III high school, Ostrów Wielkopolski	51.65	17.81	132.9
High school “Pryzmat”, Pruszków	52.16	20.79	97.5
XIX high school, Warsaw	52.24	21.07	82.6
The complex of Economic and Service Schools, Żychlin	52.18	18.26	107.7

During the instruction webinar, the teenagers were asked to record their observations when they were actually outside. They were also asked to make observations as often as possible. They were provided with a Google form with detailed instructions on how to complete the survey. The following data was collected: date, school name (so that the teacher can be contacted in case of problems with the questionnaire), age, weight, height, skin phototype (selected from the attached list), sunscreen with its sun protection factor (SPF) if used, type of activity, head cover, amount of vitamin D supplementation and style of dress. Furthermore, the teenagers recorded the time of observation, type of cloud cover, geographical coordinates of the outdoor site, maximum daily temperature and TCO_3_ prediction from Tropospheric Emission Monitoring Internet Service (TEMIS) (https://www.temis.nl/uvradiation/nrt/uvindex.php). The survey’s example can be found in the supplementary material.

**Fig. 1:**
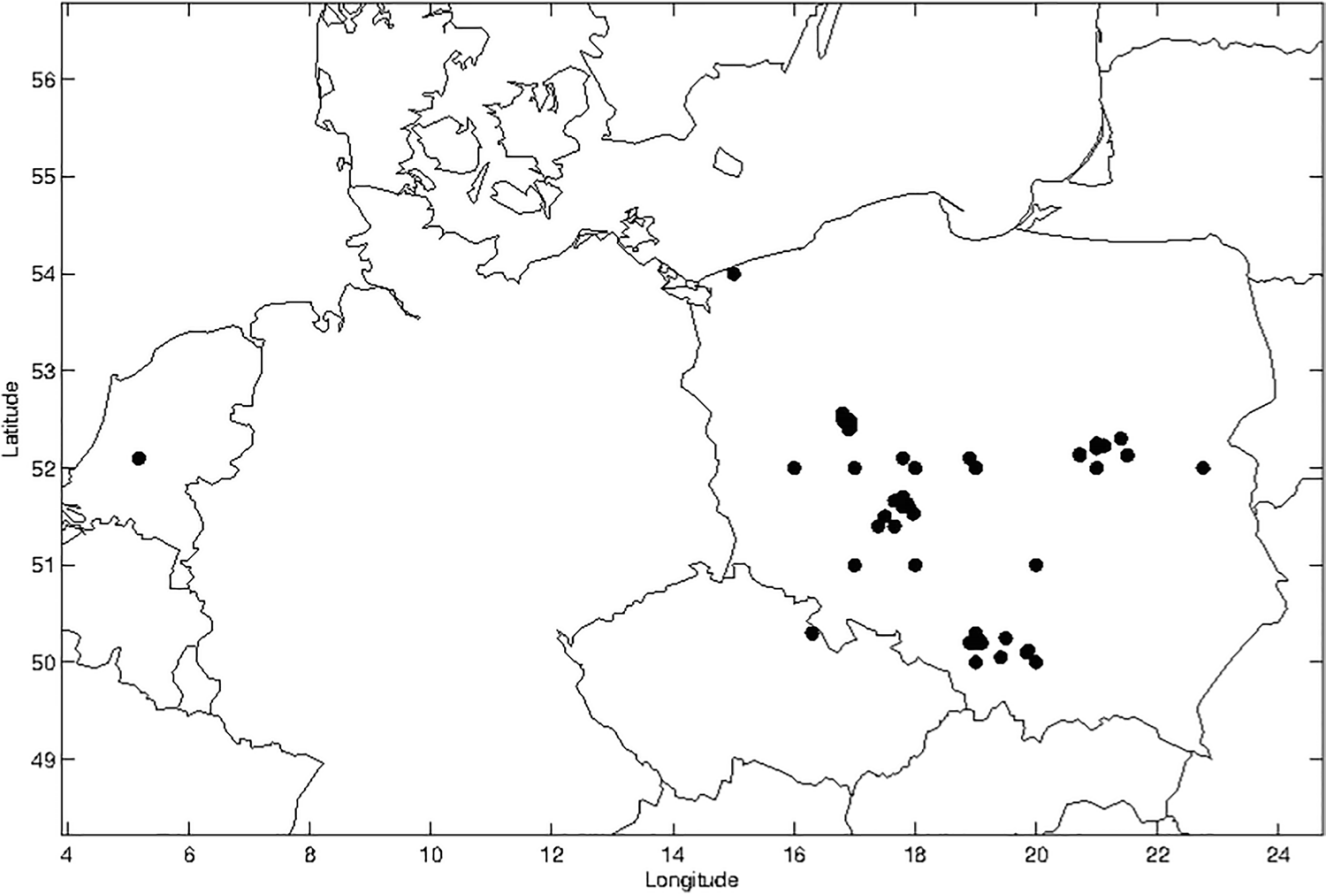
Observation sites during the BRITEC project (May–September 2020)

Most of the observations were made in the May–June period (79%), some of them were made during the summer holidays (19%) and only a few were made at the beginning of the school year in September (2%). Most sites of outdoor activities were in the vicinity of cities, not far from the schools. However, during the holiday period, the observations were made at the seaside, in mountains and even in the Netherlands. The geographical locations of the observation sites are shown in Fig. 1.

### Skin phototype

The teenagers’ skin phototypes were assessed by them on the basis of phototype characteristics described by the Fitzpatrick scale (Fitzpatrick 1988). In our study, teenagers declared the first four phototypes among the possible six types (Table 2). Skin phototype 1 was declared in 10% of surveys, 2–44%, 3–34% and 4–12%. Corresponding minimal erythemal doses (MEDs) were also taken from the Fitzpatrick study.

**Table 2:** Characteristics of the skin phototypes according to the Fitzpatrick scale (Fitzpatrick 1988)

Phototype	Characteristics	Reaction to sun	MED (J/m^2^)
1	Pale white skin, often with freckles, blue/green/hazel eyes, blond/red hair	It always burns; it is difficult to tan	200
2	Fair skin, blue/green eyes	It burns easily; it is difficult to tan	250
3	Darker white skin	It tans after the initial burn	300
4	Light brown skin	Minimal burns; it tans easily	450

Only those mentioned in the surveys are shown

### Cloudiness

Cloudiness was divided into five categories with a characteristic value of cloud modification factor (CMF), i.e. the rate of attenuation of hypothetical clear-sky UV index (UVI) by clouds. Cloud type at the observation site was assigned by the teenagers according to the possible options (Table 3) proposed by Guzikowski et al. (2017). In order to assess the accuracy of the selection of the cloud types, the differences between clouds’ categories assigned by the project participants in the same location and almost at the same time (difference between observations no more than 15 min) were compared. In 93% of the cases, the difference between the categories was not greater than 1; hence, it seems that the choice of cloud types was correct and could be used for further UV modelling.

**Table 3:** Categories of cloudiness and corresponding cloud modification factor (CMF) based on the Guzikowski classification (Guzikowski et al. 2017)

Category of cloudiness	CMF
1. Cloudless or few clouds	1.0
2. Scattered clouds	0.8
3. Broken clouds	0.7
4. Almost overcast (few blue areas)	0.5
5. Overcast	0.35

### Area of uncovered skin and body surface area

Area of uncovered skin (AUS) was calculated on the basis of the Lund and Browder chart (Lund and Browder 1944). The participants were asked to choose one of the clothing categories from the list provided. The clothing categories and the corresponding AUS values (for younger and older teenagers) are shown in Table 4. The participants could also answer “other” and describe their clothing. Four of them described their clothing as a “swimming suit”, which corresponds to ~ 70% AUS according to the Lund and Browder chart.

**Table 4:** Area of uncovered skin (AUS) as a percentage of the total body for younger (aged 11 to 15) and older (aged 16 to 18) teenagers estimated using the Lund and Browder diagram (Lund and Browder 1944)

Categories of clothing	AUS (11–15 years)	AUS (> 15 years)
Long sleeves, long trousers/skirt	11.5	10.5
T-shirt, long trousers/skirt	17.5	16.5
Sleeveless shirt, long trousers/skirt	25.5	24.5
Long sleeves, knee-length trousers/skirt	24.5	24.5
T-shirt, knee-length trousers/skirt	30.5	30.5
Sleeveless shirt, knee-length trousers/skirt	38.5	38.5
Long sleeves, mid-tight trousers/skirt	33.5	34
T-shirt, mid-tight trousers/skirt	39.5	40
Sleeveless shirt, mid-tight trousers/skirt	47.5	48

Older teenagers were treated as adult persons

### Type of activity

Geometry conversion factor (GCF) is a fraction of ambient UV irradiation that can affect the human body. It depends on the type of outdoor activity, i.e. the specific orientation of the sun on exposed parts of the body and the shading of certain areas of skin (Schmalwieser and Siani 2018). For the random position towards the sun, it was estimated as 0.46 (Pope and Godar 2010). Participants were asked to select their actual activity type from a list of possible activities or describe other activities not mentioned in the list. Table 5 shows the list of activities and corresponding GCF values, which were found in the literature (Downs and Parisi 2008, 2012; Weihs et al. 2013; Vanos et al. 2017; Schmalwieser and Siani 2018). GCF will be used for the estimation of erythemal and pre-vitamin vitamin D doses that could be obtained by the project volunteers.

**Table 5:** Geometry conversion factor (GCF) for various types of outdoor activity

Type of activity	GCF
Walking	0.5
Running	0.3
Staying	0.5
Sitting	0.6
Lying	0.7
Active tanning	0.8
Cycling	0.35
Other (random position)	0.46

### UV index and erythemal doses

The daily course of hypothetical clear-sky UVI was reconstructed by Allaart et al. (2004) model for each site provided by the project participants. The only inputs to this model were the geographical coordinates of each site (from the student surveys) and pertaining around noon TCO_3_ values, which were available for any location over the globe using the search tool from the website https://www.esrl.noaa.gov/gmd/grad/neubrew/SatO3DataTimeSeries.jsp. In case of missing TCO_3_ value, the TEMIS values collected by the students for the given location and date were used.

**Fig. 2:**
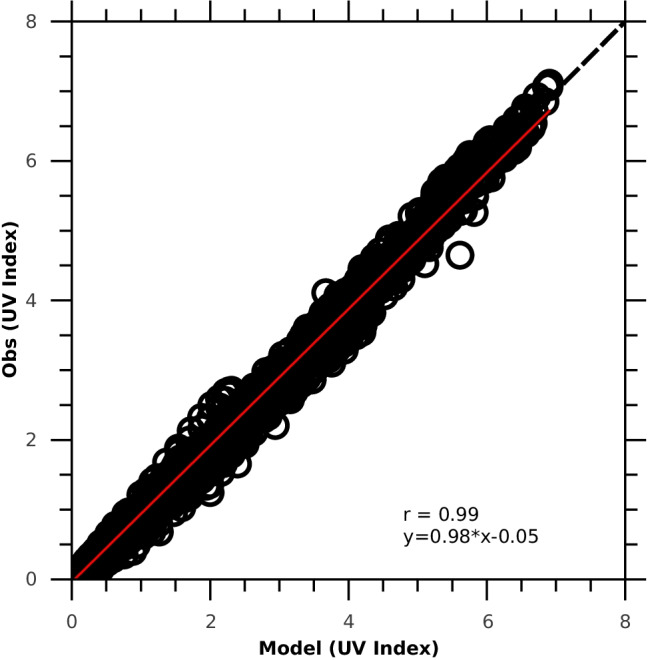
Cloudless-sky UV index measured by the Brewer Spectrophotometer at Belsk, Poland, versus corresponding values by the model of Allaart et al. (2004) for the period 2010–2015

To support the usefulness of UVIs by the model of Allaart et al. (2004) for the dose calculations, UVIs from the cloudless-sky measurements by the Brewer Spectrophotometer (BS) at Belsk (51.84° N, 20.78° E), Poland, were compared with the corresponding modelled UVI values for the period 2010–2015. Every month, the Belsk’s BS was calibrated with a set of calibration lamps. In addition, almost every 2 years, it was compared with the BS travelling world standard, which was provided by International Ozone Services, Inc. (http://www.io3.ca/Calibrations). Figure 2 presents the scatter plot of the measured UVI at Belsk versus the modelled UVI values. There is a good agreement between the BS measurements and modelled UVIs by the model of Allaart et al. (2004); i.e. the correlation coefficient was 0.99, while the linear fit line is as follows: *y* = 0.98*x* − 0.05. For all compared data, the bias was − 10.5%, but − 8.7% for the period from May to September. It seems that bias was due to the lack of the observed aerosol data in the model simulations and the BS measurement error of a few percentages (de Backer et al. 2001).

To calculate all-sky UVI values, clear-sky modelled UVIs were multiplied by CMFs from teenagers’ surveys. Guzikowski et al. (2017) found that all-sky UVIs obtained in this way, i.e. based on the model of Allaart et al. (2004) and cloud observations by non-meteorologists, had a better agreement with UVI measurements than predictions of all-sky UVI using an advanced numerical model with detailed cloud forecasts. The risk of overexposure was obtained for each project participant by comparing the time spent outside with the minimum time needed to obtain redness of the skin, which is counted from the beginning of the stay outdoors. Teenagers were asked to make observations only during their outdoor activities. Thus, the duration of the solar exposure was calculated as the time difference between the first and the last observations while continuously spending time outdoors. In the case of recording only one observation per day, we assumed that the time spent outdoors was 15 min, which was the recommended minimum interval between observations.

### *Vitamin D*_*3*_* doses*

Personal vitamin D doses were calculated based on the model (Eqs. (1)–(3)) used in our previous articles (Guzikowski et al. 2018; Czerwińska and Krzyścin 2020a; Czerwińska and Czuchraj 2021):

1 $$Q_{\;VitD_3k}\left(t_0,\Delta t\right)={Rate\;}_k\cdot{VitD}_{3,P}\left(t_0,\Delta t\right)\cdot ESA\cdot AF$$

2 $${VitD}_{3,P}\left(t_0,\Delta t\right)={VitD}_{3,A}\left(t_0,\Delta t\right)\cdot GCF$$

3 $${VitD}_{3,A}\left(t_0,\Delta t\right)=\int_{t_0}^{t_0+\Delta t}a\left(SZA,{TCO}_3\right)\cdot UVI_{clear}\cdot CMFdt$$

where $Q_{{{\text{VitD}}_{{3}} ,k}} \left( {t_{0} ,\Delta t} \right)$ is the amount of vitamin D from skin synthesis in the period from *t*_0_ to *t*_0_ + Δ*t*, equivalent to vitamin D from oral supplementation in IU; Rate_*k*_ is equal to 6153 IU/MED_*k*_ is the vitamin D production rate (amount of vitamin D equivalent to oral supplementation in IU due to the pre-vitamin D exposure of 1 J per 1 m^2^ skin area) depending on skin phototype “*k*” (from 1 to 4) with the corresponding MED_*k*_ value; VitD_3,P_(*t*_0_,Δ*t*) is a personal pre-vitamin D_3_ dose in J/m^2^ obtained during the Δ*t* interval; ESA is a geometrical area of the exposed skin in m^2^; and AF is an age factor (accounting for the loss of ability to synthesize vitamin D with age, AF = 1 for children and young adults; MacLaughlin and Holick 1985). ESA was calculated as the uncovered part of the total body surface area (BSA) in m^2^ depending on the way of clothing (according to Table 4), i.e. ESA = AUS·BSA. BSA values were estimated from the weight and height of the project persons using the Mosteller formula (Mosteller 1987).

VitD_3,P_(*t*_0_,Δ*t*) is approximated as a product of the ambient pre-vitamin D dose, VitD_3,A_(*t*_0_,Δ*t*) and GCF (Eq. (2)). Formally, VitD_3,A_(*t*_0_,Δ*t*) is the time integral of the pre-vitamin D irradiance over the period of outdoor activity. Here, the pre-vitamin D irradiance is estimated from all-sky UVI (UVI_clear_·CMF) using the erythema to pre-vitamin D conversion factor *α* depending on solar zenith angle (SZA) and TCO_3_ (Eq. 3). The conversion factor was determined experimentally by analysing the Brewer Spectrophotometer spectra at Belsk (Czerwinska and Krzyścin 2020b).

To summarize, the calculation of the personal skin-synthetized vitamin D dose, which is equivalent to the amount of vitamin D taken orally, requires personal data (ESA, GCF, AUS, period of being outside and skin phototype), geographical coordinates for the exposure site and the geophysical data (cloudiness level and TCO_3_). Almost all input values for our model (Eqs. (1)–(3)) were inferred from the surveys. Only ozone data was taken from external resources from the website.

## Results

### Area of uncovered skin

**Fig. 3:**
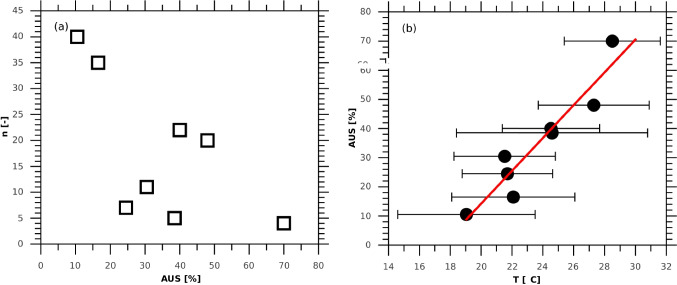
**A** Number of the surveys reported for each category of AUS according to Table 4. **b** AUS as a function of the air temperature. The red line represents the linear regression line of AUS on ambient temperature. The horizontal bars are the mean value of AUS ± standard deviation

Most of the teenagers’ AUSs were in the range of 10.5–17.5% (long sleeves or T-shirts and covered legs, Table 4), but also those with AUS of ≥ 40% (T-shirt and mid-tight trousers/skirt) were identified in 44 surveys (Fig. 3a). Figure 3b shows the relations between AUS and the maximum daily temperature. An apparent increase of AUS with air temperature was found; i.e. the regression line of AUS on ambient temperature appeared statistically significant, which is in line with previous studies (Chubarova et al. 2013; Guzikowski et al. 2018).

**Fig. 4:**
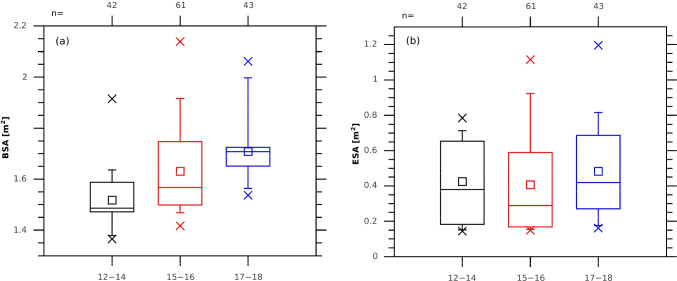
Box plot illustrating the statistic distribution of the data for the three age groups (12–14 years old, 15–16 years old and 17–18 years old). **a** BSA. **b** ESA. Edges of the boxes show 25/75 percentile, whiskers 5/95 percentile and crosses the maximum/minimum value. The horizontal line is the median, and the small square is the mean value of the sample

BSA was calculated for each survey, from the recorded height and weight of the participant, using the Mosteller formula (Mosteller 1987). The mean value was 1.62 m^2^, with a minimum of 1.4 and a maximum of 2.1 m^2^. Analysis of BSA and ESA for different age groups (12–14 years old, 15–16 years old and 17–18 years old) revealed an expected increase of BSA with age, but this was not the case with ESA (Fig. 4). The youngest (the oldest) teenagers exposed a larger (smaller) part of the body, which compensated for their smaller (larger) BSA.

### Risk of overexposure

Two behavioural patterns were considered in the calculations of the UV exposure:

- Horizontal parts of the body (e.g. arms and ears) were exposed at all times while staying outdoors
- Parts of the body were temporarily shaded during outdoor activities.

**Fig. 5:**
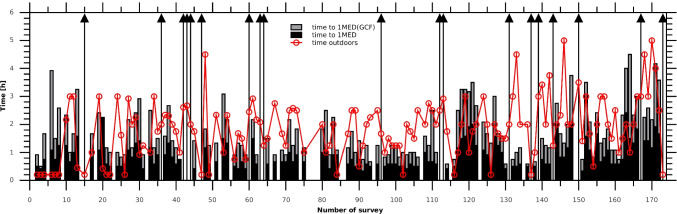
Time needed to obtain 1 MED by exposing horizontal parts of the body (black bars) and during various activities characterized by a GCF value from Table 5 (grey bars). The red line is the time spent outdoors provided by the teenagers in the surveys

**Fig. 6:**
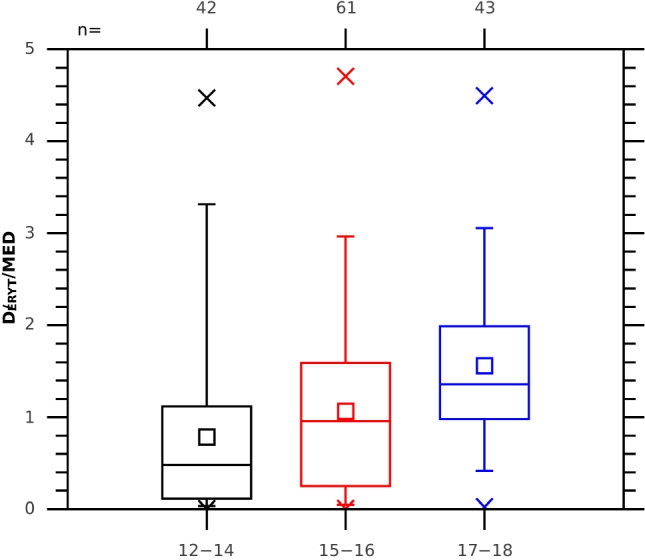
Box plots for the normalized (divided by 1 MED) erythemal doses (obtained during various outdoor activities listed in Table 5) obtained during the day in the three groups of the project participants. See Fig. 4 for the box plot notation

Thus, ambient and personal (weighted by GCF according to various types of activities, Table 5) erythemal doses obtained by the teenagers were analysed, respectively, to estimate the time needed for the erythema occurrence. The majority of the teenagers reported walking as their outdoor activity. Figure 5 shows the times needed to exceed 1 MED using both of the above-mentioned behavioural patterns. We also divided teenagers into three age groups: 12–14 years old, 15–16 years old and 17–18 years old. The distribution of GCF values in each group, i.e. types of outdoor activities, was almost similar. The increase of erythemal overexposure with age is illustrated in Fig. 6.

### Vitamin D_3_

**Fig. 7:**
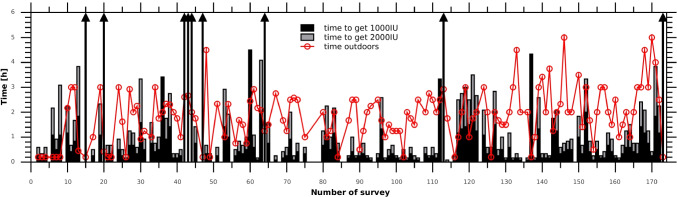
Time needed to get 1000 IU (black bars) and 2000 IU (grey bars) of vitamin D due to skin synthesis. The red line is the time spent outdoors by the teenagers

Vitamin D dose equivalent to oral supplementation was estimated from numerical calculations only (Eqs. (1)–(3)); i.e. no blood tests were performed. Figure 7 shows the time needed to obtain the dose equivalent to 1000 IU (2000 IU) of oral supplementation compared with the time spent outdoors declared by the participants.

**Fig. 8:**
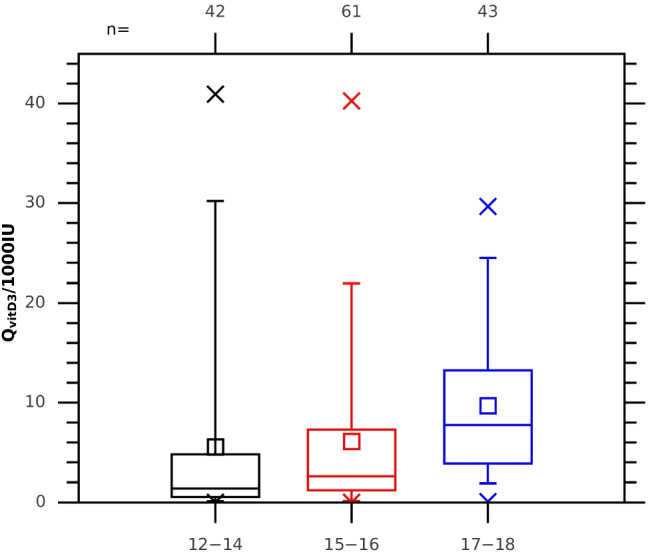
The same as Fig. 6 but for the normalized (divided by 1000 IU) vitamin D doses equivalent to the oral supplementation

Similarly, as in the case of the daily erythemal dose estimations, we found that there are differences in vitamin D skin synthesis according to the age groups (Fig. 8). It is worth mentioning that a large number of teenagers obtained high doses in the youngest group as the mean dose was larger by ~ 2000 IU than the median. The difference between the mean value and median was also found in older teenagers. The smallest difference (~ 1000 IU) was in the oldest group. In each group, there were students (~ 5% of the population) who received daily doses in excess of 20,000 IU.

### Uncertainty of the dose estimations

The limitation of the study, which can affect the uncertainty, has been identified as the lack of verification of the participants’ decision as to their phototypes and the type of cloud cover over the tanning site. For many students, the distinction between phototypes II and III or between scattered and broken clouds may not have been obvious. This could lead to uncertainties in the results. For example, if phototype III was wrongly selected instead of phototype II, the vitamin production rate (Eq. (1)) would be 20% lower. Therefore, we conducted a test to estimate the uncertainty range of the dose calculations. This was done by repeating the calculations after switching between these two phototypes and cloud types; i.e. the reported phototype II (III) was replaced with phototype III (II) and the same procedure was used for cloud observations if scattered and broken clouds were reported.

Table 6 shows the mean erythemal and pre-vitamin D doses for each age category based on the original data (from the surveys), data after the switching procedure used for phototype II/III, scattered/broken clouds and both factors simultaneously. The differences between the daily doses were a maximum of ~ 10% for the same age category. Moreover, the percentage of exceeding the thresholds (MED, 1000 IU and 2000 IU) differed only slightly from the original data. Thus, the final conclusions are consistent for all considered scenarios.

**Table 6:** Daily mean erythemal (in personal MED values) and pre-vitamin D doses equivalent to the oral supplementation (in 1000 IU) for three age categories (12–14 years old, 15–16 years old and 17–18 years old)

Age category (years)	Original Data		MED Switch		Cloud Switch		MED&Cloud Switch	
	< *D*_ERYT_ > [MED]	< *Q*_VITD3_ > [1000 IU]	< *D*_ERYT_ > [MED]	< *Q*_VITD3_ > [1000 IU]	< *D*_ERYT_ > [MED]	< *Q*_VITD3_ > [1000 IU]	< *D*_ERY_ > [MED]	< *Q*_VITD3_ > [1000 IU]
12–14	0.78	5.56	0.86	6.29	0.78	5.53	0.86	6.26
15–16	1.07	6.10	1.05	5.99	1.05	6.03	1.03	5.90
17–18	1.56	9.68	1.53	9.36	1.56	9.67	1.52	9.35

The doses are calculated using Eqs. (1)–(3) with the input taken from the survey data (“Original Data”) and after the switch: MED II ↔ MED III (“MED Switch”), broken ↔ scattered clouds (“Cloud Switch”) and MED II ↔ MED III and broken ↔ scattered clouds (“MED&Cloud Switch”).

Also, we did not include the possibility of having vegetation present into our estimations as we only considered the worst-case scenario for the erythema appearance which comprises no shade due to vegetation. If the presence of vegetation was included, the doses would be smaller.

## Discussion and conclusions

In Poland, during the school year, children have a limited number of opportunities to spend time outdoors during the school day. Young children have time in the playground included in their daily schedule. Teenagers have a very busy study schedule, over 35 h a week. For 4 h a week, teenagers have PE lessons, but only a part of this time is spent in the schoolyard—most of it is spent in the gym hall. Thus, a typical teenager can only spend time outdoors after the lessons, i.e. after 3 p.m. At this time, the ultraviolet radiation intensity, especially from September to April, is not enough to obtain the recommended dose of vitamin D due to the skin synthesis. The only time to improve students’ vitamin D status is during holidays, weekends and school trips (typically in spring and early autumn). Farrar et al. (2016) found out that teenagers (12–15 years old) failed to provide adequate vitamin D levels due to sun exposures, even though children in the UK spend more time in schoolyards during breaks and PE lessons. Also, Al-Othman et al. (2012) observed that among Saudi youth, vitamin D status improved by promotion of an active lifestyle and outdoor activities, but the vitamin D deficiency remained.

Teenagers aged 12 to 18 years old are a group, in which the deficiency of vitamin D is most often observed (Gonzalez-Gross et al. 2011Al-Othman et al. 2012; Vierucci et al. 2013; Płudowski et al. 2016; Farrar et al. 2016 Kunz et al. 2018). Nevertheless, while they usually do not spend enough time outside, they exhibit inadequate sun exposure patterns (Fernández-Morano et al. 2017; Hubbard et al. 2020; Liew and Cust 2021). Fernández-Morano et al. (2017) found that even though teenagers have sufficient knowledge of sun risk and the importance of sunscreen use, they do not care about solar overexposures and still suffer from sunburns. In Australia, there were many social campaigns on safe staying outdoors, but skin cancer incidence rates have been rising rapidly. Liew and Cust (2021) assessed the change in the patterns of outdoor exposure among the whole age population in Australia for the period from 2003 to 2016. They found that in the group aged 12–17, there was no change in the consciousness of the tanning risk, in contrast to the other age groups. Therefore, social campaigns should be specifically targeted at young people. Patterns of risky behaviour among teenagers are common around the world and also existed before the first COVID-19 lockdown in 2020 (Fernández-Morano et al. 2017; Hubbard et al. 2020; Liew and Cust 2021).

In Poland, during the first months of the pandemic in 2020 (March–April), there were heavy restrictions that recommended staying at home for as long as possible. Online learning was ordered, and schools remained closed until the end of the school year in June. Since May, children were allowed to go outside in their free time. Therefore, estimates of the erythemal and vitamin D doses were possible for periods of teenagers’ outdoor activities in their free time.

The analysis comprises results of 146 surveys on patterns of outdoor activity recorded by 12–18-year-old volunteers. Although the teenagers could obtain the recommended dose of vitamin D (between 800 and 2000 IU; Rusińska et al. 2018), they tend to put themselves at risk when tanning. Less than 15% used sunscreens with SPF between 15 and 50, and only ~ 18% covered their heads.

We found that 67% of the surveys showed risky behaviour with daily supra-erythemal doses (> 1 MED) assuming exposure of horizontal body parts, compared with 48% when some part of the body could be shaded during declared outdoor activities according to the options in Table 5. The risk of overexposure to UV radiation increased with age, despite the anticipated increase in awareness of the harmful effects of UV radiation. In 26% of the questionnaires completed by younger teenagers (12–14), supra-erythemal doses (> 1 MED) were obtained, in 46% for those aged 15–16, and as many as 72% for the oldest ones (17–18). The Wilcoxon test concluded that the differences between these groups were statistically significant. The youngest children were more likely to have been supervised by the adults. Unfortunately, we did not count how many of the participants indeed suffered from sunburn.

The number of volunteers (~ 100) appears to be large enough to reveal differences in the risk of overexposure between the considered age groups. This is also supported by the proposed method of calculating the uncertainty of personal doses, which takes into account the possibility of incorrect selection of the phototype and cloudiness level by the volunteers. It is worth mentioning that the frequency of phototypes declared by the volunteers, i.e. mainly II and III and the rare occurrence of phototype I and IV, corresponds to other studies on the frequency of phototypes in Poles and for other European countries (van der Leest et al. 2011; Sitek et al. 2016; Bieliauskiene et al. 2020).

Teenagers were eager to go outside when it became possible to do so, after 2 months of staying home during the first lockdown (March–April 2020). For many of them, there was practically no time limit for outdoor activities. At times, exposures lasting longer than 4 h were recorded. In all age groups, ~ 5% of the surveys showed very high overexposures of ~ 3 MED. This behaviour could not have happened in a typical school year. Overexposures were expected during the summer holidays (especially for older teenagers). However, by this time, the skin was already partly prepared for high UV radiation, as teenagers previously had the opportunity to expose their skin during spring. In 2020, the teenagers only started venturing outside in May when the UV radiation was usually strong, but at that time, their skin was not adapted to high surface UV radiation.

Our calculations of the vitamin D doses that could be gained by teenagers due to skin synthesis show a large potential to improve their vitamin D status. The majority of the teenagers could obtain the daily vitamin D dose (equivalent to oral supplementation) of over 1000 IU (in 77% of the surveys) and 2000 IU (in 66% of the surveys). For some teenagers, the vitamin D dose exceeds a few times the recommended daily dose. In the first group (12–14 years old), 57% (43%) could obtain the dose equivalent to oral intake over 1000 IU (2000 IU); in the second group (15–16 years), 75% (62%); and in the oldest group (17–18 years), more than 90%. However, prolonged sunbathing is not effective for vitamin D production as only a part of the obtained pre-vitamin D dose could be converted to 25(OH)D (i.e. used to improve vitamin D status). In such cases, other inactive metabolites (e.g. lumisterol) are produced, lowering the potential of pre-vitamin D to increase vitamin D levels in the body (e.g. Acar and Özkan 2021). Therefore, striving to obtain high doses of pre-vitamin D despite the risk of erythema is not a good strategy to strengthen the immune system, which is of special importance in the COVID-19 era.

Solar exposures to sub-erythemal doses (< 1 MED) could also improve the vitamin D status. Based on the survey results and assuming that the supervised sun exposures by the teenagers were aimed at receiving safe doses, i.e. between 0.5 and 1.0 MED, we found that 76–86% (44–79%) of them could obtain a pre-vitamin D dose above 1000 IU (2000 IU). It is worth mentioning that tools to control the duration of tanning at the site of outdoor activities have already been prepared (Guzikowski et al. 2017, with the dose control application from the website http://cirrus.cba.pl/erythema/).

To improve vitamin D levels without the risk of sunburn, teenagers’ self-awareness needs to be changed. The time outdoors should be controlled. Furthermore, the most exposed parts of the body (e.g. arms, ears, forehead) should be protected (covered or sunscreen applied). Similarly, to Fernández-Morano et al. (2017), we noticed that even if our participants had the knowledge of photoprotection (the researcher gave online lectures for project participants on the risk of sun exposure, in a non-scientific language), they did not put it into practice. Therefore, we agree that social campaigns should be targeted at this age group. Perhaps the solution would be to present amusing educational videos on social media (Facebook, Instagram and TikTok) and/or smartphone apps. During the BRITEC project, some of the students were very interested in the subject and wanted to share their newly gained knowledge with younger children from their school (Complex of Economic and Service Schools in Żychlin). If hearing about the risks of overexposure from adults and authorities does not seem to work with adolescents, perhaps messages from their peers will be more effective.

## Supplementary Information

Below is the link to the electronic supplementary material.

Supplementary file1 (DOCX 15.5 KB)
