# Supplementary material for: Exposure to solar UV radiation of Polish teenagers after the first COVID-19 lockdown in March–April 2020
Source: Int J Biometeorol. 2022 Aug 1;66(10):2021–32. doi: 10.1007/s00484-022-02337-8 (PMC9340691; doi:10.1007/s00484-022-02337-8)
Supplement: Supplementary file 1 — Supplementary file1 (DOCX 15.5 KB) [file 484_2022_2337_MOESM1_ESM.docx]

Questionnaire UV radiation (I part). Data needed for vitamin D dose calculation.

1. School and class (required)
2. Age (required)
3. Weight (required)
4. Height (required)
5. Current date (required)
6. Maximum daily temperature (required)
7. Select the category of clothing, which the best describes your outfit. If your outfit does not match any of the categories, describe it in the position “other” (required):

- Long sleeves, long trousers/skirt;
- T-shirt, long trousers/skirt;
- Sleeveless shirt, long trousers/skirt;
- Long sleeves, knee-length trousers/skirt;
- T-shirt, knee-length trousers/skirt;
- Sleeveless shirt, knee-length trousers/skirt;
- Long sleeves, mid-tight trousers/skirt;
- T-shirt, mid-tight trousers/skirt;
- Sleeveless shirt, mid-tight trousers/skirt;
- Other (describe).

1. Do you have a head cover? If you have, select from below:

- Baseball cup;
- Wide breamed hat;
- Piece of cloth.

1. Which of the phototypes the best suits you (required)?

- I Pale white skin, often with freckles, blue/green/hazel eyes, blond/red hair. It always burns, it is difficult to tan;
- II Fair skin, blue/green eyes. It burns easily, it is difficult to tan;
- III Darker white skin. It tans after the initial burn;
- IV Light brown skin. Minimal burns, it tans easily;
- V Brown skin. Rarely burns, easily tans;
- VI Dark brown/black skin. Never burns, always tans.

1. Did you use sunscreen? If you do, which SPF did you use (required)?
2. Do you supplement vitamin D? If you do, how much IU (International Units) per day?
3. Select the type of activity, that you were conducting for the most of the time during outdoors. If your activity does not match any of the categories, describe it in the position “other” (required):

- Walking;
- Running;
- Staying;
- Sitting;
- Lying;
- Active tanning;
- Other (describe).

1. Total ozone amount and predicted UVI in your site (from <http://temis.nl/uvradiation/nrt/uvindex.php>) (required).
2. Your site geographical location (latitude and longitude) (required).

Questionnaire UV radiation (II part). Observations.

Insert UVI from forecast (<http://meteoweb.pl/nowcasting>) or Android application (<http://meteoweb.pl/sun>). If you have your UVI meter, you can use it here. There is a possibility to insert 12 observations per day. If there is less observations, select an option “end for today”. The most important are the observations between 11am to 2pm (local time).

1. The source of UVI, you are using (required):

- www forecast (<http://meteoweb.pl/nowcasting>);
- Android application (<http://meteoweb.pl/sun>);
- Other (describe).

1. Hour of the observation (required)
2. UVI (cloudless sky from forecast or app) (required)
3. Category of cloudiness (required):

- Cloudless or few clouds;
- Scattered clouds;
- Broken clouds;
- Almost overcast (few blue areas);
- Overcast.

1. UVI (including cloudiness) (required)
2. Was it the last observation for today (required)?

- End for today
- No (redirection to the new observation form)
